# Multilevel Job Demands and Resources: Cross-Level Effects of Competing Organizational Facet-Specific Climates on Risky Safety Behaviors

**DOI:** 10.3390/ijerph17103496

**Published:** 2020-05-17

**Authors:** Valerio Ghezzi, Tahira M. Probst, Laura Petitta, Claudio Barbaranelli

**Affiliations:** 1 Department of Psychology and Sapienza, University of Rome, 00146 Rome, Italy; laura.petitta@uniroma1.it (L.P.); claudio.barbaranelli@uniroma1.it (C.B.); 2 Department of Psychology, Washington State University (WSU), Vancouver, WA 98686, USA; probst@wsu.edu

**Keywords:** multilevel modeling, organizational production pressure climate, organizational safety climate, risky safety behaviors, workload

## Abstract

Both individual demands (i.e., workload) and organizational demands and resources (i.e., production pressure and safety climates) may affect the likelihood that employees undertake risky safety behaviors in different ways. Adopting an organizational multilevel perspective, the aim of the present research was fourfold: (1) to examine the impact of individual-level job demands (i.e., workload) on the enactment of risky safety behaviors; (2) to evaluate the effects of coexisting and competing organizational facet-specific climates (i.e., for safety and for production pressure) on the above outcome; (3) to assess their cross-level interactions with individual job demands, and (4) to test the interaction among such organizational demands and resources in shaping risky behaviors. A series of multilevel regression models tested on surveydata from 1375 employees nested within 33 organizations indicated that high workload increases the likelihood of employees enacting risky safety behaviors, while organizational safety and production pressure climates showed significant and opposite direct effects on this safety outcome. Moreover, organizational safety climate significantly mitigated the effect of individual job demands on risky safety behaviors, while organizational production pressure climate exacerbated this individual-level relationship. Finally, organizational safety climate mitigates the cross-level direct effect of organizational production pressure climate on the enactment of risky safety behaviors.

## 1. Introduction

Modern organizational contexts require the performance of a multitude of job activities and tasks in fast-paced environments. These can be experienced as extremely demanding for workers, especially if they are not adequately supported by personal and/or organizational resources. According to the job demands-resources (JD-R) model, job demands are defined as “physical, psychological, social, or organizational aspects of the job that require sustained physical and/or psychological effort” [1] (p. 274), whereas job resources refer to “physical, psychological, social, or organizational aspects of the job that are functional in achieving work goals [and] reduce job demands” [1] (p. 274). One of the core underpinnings of the JD-R model is that negative effects of job demands on undesirable individual and organizational outcomes are likely to increase if not adequately mitigated by individual or organizational job resources [1,2]. Moreover, organizational demands may accumulate with those at the employee level, therebyaccruing their negative effects on undesirable outcomes [1]. Although there is a plethora of theoretical and empirical contributions highlighting the role of individual resources and demands (and their interplay) in explaining employee wellbeing [3] and behaviors within the organization [4], the role of organizational-level resources and demands has been much more rarely investigated [5].

Excessive job demands can have serious implications for employee safety outcomes [6,7]. For example, research argued that cognitive and physical workload can negatively affect safety compliance [8], namely the adherence to safety rules and practices within the workplace [9]. Moreover, ignoring safety rules or enacting risky behaviors at work can be viewed as proximal antecedents of a number of negative safety outcomes [7], such as injuries and accidents [10]. It is worth noting, however, that job demands and resources are not confined to the individual sphere [5]. Indeed, they may also exist at higher levels of the organizational system (e.g., organizational facet-specific climates [11]), and they may contribute to shaping individual differences in the enactment of safety behaviors in many ways. For example, organizational demands (e.g., production pressure climate) and resources (e.g., safety climate) may exert a direct impact on individual safe behaviors at work, but they may also interact with individual determinants (e.g., workload) of safety performance. Moreover, organizational demands and resources may also interact at their own level of analysis, and their interplay may affect employees’ safety performance by highlighting specific boundary conditions of their effects, since they operate in concert within organizational contexts rather than in isolation [1].

Given these premises, the purpose of the present research is to integrate the JD-R model and organizational climate theory within a multilevel framework for studying workplace safety. Specifically, the aim of the present study is to investigate the role of multiple organizational-level resources and demands (specifically, organizational facet-specific climates for safety and for production pressure) and their interplay in shaping individual enactment of risky safety behaviors. Moreover, the present study aims to investigate whether such organizational demands and resources may simultaneously exacerbate or attenuate the relationship between individual demands (i.e., workload) and employee safety behaviors (for a detailed flow chart of the study process, see Figure S1 of Supplementary Materials). Below, we begin by discussing the impact of workload on employee safety behaviors. Next, we develop hypotheses regarding the effects of two organizational-level variables (i.e., organizational safety and production pressure climates) on employee-level safety behaviors, how these variables may modulate the individual demand-safety relationship and how their interplay may provide added value in predicting the enactment of risky safety behaviors by employees.

## 2. Theoretical Framework and Literature Review

### 2.1. The JD-R Model and Safety Behaviors Within the Multilevel Framework

As alluded to by the JD-R model, unsafe behaviors within the workplace should not be viewed as simply thebyproduct of individual initiatives and decisions to enact a specific pattern of risky actions for one’s own physical safety [12,13]. Indeed, there are numerous organizational determinants (i.e., demands and/or resources) that can contribute to increasing or decreasing the likelihood of enacting behaviors that are at odds with principles of safety within the workplace. For example, multiple aspects of organizational climate may be viewed as conjoint determinants of individual safety compliance [14]. In line with the JD-R model and some recent calls for its extension to a multilevel conceptual framework [5,15], we propose that production pressure and safety facet-specific climates may act, respectively, as important organizational demands and resources that contribute to explain the enactment of risky safety behaviors [6,7,16]. Indeed, safety climate, namely the “[shared] individual perceptions of the value of safety in the work environment” [9] (p. 100) has been acknowledged as a paramount organizational resource for reducing the likelihood of enacting risky behaviors [17]. On the other hand, production pressure may be meaningfully operationalized at the organizational level as a facet-specific climate reflecting a shared set of job demands related to the attainment of “operational goals for the purpose of increasing organizational profits and/or efficiency” [18] (p. 581). Such perceived demands may increase the likelihood of employees undertaking risky behaviors in order to reach productivity goals more quickly and efficiently.

Although organizational safety climate resources and production pressure climate demands may directly affect individual safety-oriented behaviors within the workplace, we propose they can also interact with individual-level demands in explaining employee safety-related behavior. In line with this, research has demonstrated that organizational safety climate attenuates the impact of individual-level job stressors on different aspects of safety-related behavior at work [17,19], but less is known about the role of organizational production pressure demands in potentially exacerbating the impact of individual job demands on such behaviors [20]. Moreover, researchers contend that it is important to simultaneously consider coexisting and potentially competing organizational climates so as not to misspecify their relative importance in predicting employee behavior related to workplace safety. Yet, the vast majority of studies have focused on single facet-specific organizational climates [21,22]. Unfortunately, this approach can rarely be considered the “most productive path to creating a full and accurate understanding of how climates affect individual outcomes within organizations” [11] (p. 706), since it is very unlikely that multiple facet-specific climates do not coexist simultaneously within organizations or operate in isolation in shaping organizational behavior [23]. Moreover, it is difficult to envision an organization where demands and resources (and their interplay) do not simultaneously determine employees’ outcomes [24].

### 2.2. Individual Workload and Safety Behaviors

Workload is framed within the JD-R model as an individual-level job demand. Specifically, the term “workload” is commonly used as a very broad category referring to “any variable reflecting the amount or difficulty of one’s work” [25] (p. 222). Here, we focus on its narrow quantitative facet, namely the “perceived amount of work in terms of pace and volume” [26] (p. 358). Workload perceptions may stem from a disconnection between “objective” job requirements and one’s perceived ability to cope with them [27], and there is considerable evidence that both cognitive and physical workload perceptions vary primarily as a function of individual differences [28]. Despite subjective perceptions of workload may vary as a function of other individual characteristics (e.g., [27]), there is evidence that a high workload may compromise workplace safety, both for the employees [29] and other stakeholders (e.g., patients, [30]). Moreover, high individual demands may result in a lack of both structural and psychological empowerment [31], which are important determinants of individual safety within the workplace [32].

However, while much attention has been devoted to investigating the impact of individually perceived workload on well-being [33,34] and job performance [35,36], empirical evidence concerning the workload-safety link is surprisingly limited. For example, Hansez and Chmiel [13] argued that an increase in job demands may lead to systematic or routine violations of safety rules due to compensatory processes aimed at maintaining stable job performance outcomes in the face of excessive job demands [37]. Specifically, an employee experiencing a high level of workload may disengage from safety compliance by enacting behaviors at odds with safety requirements in order to reach productivity goals [38]. Given these premises, we expect that:

**Hypothesis** **1.** *Individual workload is positively related to the enactment of risky safety behaviors.*

### 2.3. Multiple Organizational Facet-Specific Climates and Safety Behaviors

Organizational climate refers to the observable attitudes and behaviors of organizational members [39] regarding practices, procedures, and rewarded behavior [40]. Although it was originally conceptualized as a “molar” construct [41], research has increasingly focused on organizational facet-specific climates in relation to specific targets of employees’ perceptions, giving rise to the so-called “climate for” approach [42], in which employees are asked to report their perceptions towards specific aspects of the organizational policies, practices and rewarded behaviors (e.g., safety, production, justice, customer service). The aggregation of such perceptions within the organization represents the classical operationalization of the underlying organizational facet-specific climate [43].

Studies focusing on facet-specific climates have provided a valuable contribution in several fields of organizational and health psychology. For example, a large body of evidence converges in highlighting safety climate as a key determinant of employees’ safety perceptions and behaviors within the workplace [6,7,10,17,19]. However, one of the limitations of the “climate for” approach is that researchers have often examined the impact of facet-specific climates only in relation to facet-specific outcomes, that is narrow outcomes tied to specific facets of organizational climate [11], such as injuries and accidents as outcomes of organizational safety climate. In a similar vein, the interactive (cross-level) effects of facet-specific climates on the relationship linking individual determinants to safety performance have been frequently examined only considering single facet-specific climates (although there are rare exceptions [20,44]).

Despite the dearth of research incorporating multiple climates, researchers have acknowledged the theoretical and practical importance of simultaneously considering multiple facet-specific climates (e.g., [45,46,47]). Such an approach provides three distinct advantages above a single facet-specific climate approach. First, it encourages researchers to model simultaneously multiple aspects of the molar organizational climate that generally coexist and operate in concert. For example, Jiang and Probst [20] found that both group safety and productivity climates affect safety compliance (with opposite directions of their relative effects). Second, it allows researchers to examine how different organizational demands and resources mitigate (or exacerbate) the impact of individual resources and demands on facet-specific or non-facet specific outcomes. In the same study as above, Jiang and Probst [20] found that the impact of the safety-production conflict (SPC) on accident reporting attitudes was simultaneously attenuated by customer service climate and exacerbated by group-level productivity climate. Third, it provides a rationale to examine the boundary conditions of the effects of multiple climates at different levels of analysis (e.g., organizational and/or individual). For example, Myer et al. [23] found in their research a positive significant interaction among organizational service and ethical climates on organizational financial performance but no significant main effects.

In the present study, we focus on the role of two organizational facet-specific climates (i.e., for safety and for production pressure) representing, respectively, two pivotal organizational resources and demands potentially competing for the enactment of a consistent pattern of behavior from the employees (i.e., safe versus unsafe behavior).These constructs are of key interest for organizational climate theory focused on workplace safety, since they underlie the basic elements of safety-production compatibility systems [48], where employees are required to work under production pressure without avoidingto behave safely. However, if organizational demands regarding production pressure exceed safety climate resources, this may seriously undermine employees’ health and safety. Moreover, in cases of relevant mismatches between organizational safety resources and production pressure demands, employer’s goals may conflict with those of the employees [49], and this may result in ambiguous appraisals of organizational policies and practices potentially undermining employee safety

#### 2.3.1. Organizational Safety Climate and Safety Behaviors

Shared perceptions of safety within organizational contexts reflect the extent to which safety is rewarded, expected, valued and reinforced by the organization [50]. Interestingly, although previous empirical research has operationalized safety climate both at the individual-level (i.e., psychological safety climate perceptions [51]) and at higher contextual levels (i.e., shared perceptions at the workgroup or organizational level [52]), Christian et al. [6] found that the higher-level operationalizations of safety climate demonstrated stronger relationships with individual safety outcomes than those found with psychological safety climate. Accordingly, in our current study we operationalize safety climate at the organizational level.

Using the JD-R model as our theoretical foundation, we propose that a positive organizational safety climate can be viewed as a beneficial organizational-level resource for employees. Indeed, recent meta-analyses summarizing the extant work has found that a positive safety climate is predictive of a host of beneficial employee safety-related outcomes, including improved safety knowledge, safety motivation, compliance, and safety participation, as well as reduced accidents and injuries (e.g., [6,7,17]). Based on these meta-analytic research findings, we expect that:

**Hypothesis** **2.** *A positive organizational-level safety climate is predictive of fewer risky safety behaviors enacted by employees.*

Moreover, we argue that the resource of a positive safety climate not only has direct positive benefits (i.e., a main effect) on employee safety, but will also attenuate the negative effects of job demands facing employees. In particular, under conditions of high workload, a positive organizational safety climate may provide employees with secondary support mechanisms [53], allowing them to better meet those demands without compromising safety. For example, a positive safety climate may signal to employees that it is acceptable to maintain appropriate safety behaviors even under conditions of high job demands, because such behaviors are normatively valued and rewarded by the organization. On the other hand, under conditions of a poor safety climate, employees facing high workload demands may perceive a low organizational value for safety and engage in risky safety behaviors in order to meet those demands. Thus, we expect the following cross-level interaction effect:

**Hypothesis** **3.** *A more positive organizational safety climate significantly attenuates the impact of individually perceived workload on the enactment of risky safety behaviors.*

The paths leading to a given level of job performance may substantially vary across organizations (i.e., the organizational equifinality principle, [54]). For example, organizations exhibiting high production standards may undervalue safety aspects [52]. In this sense, safety and productivity can be viewed as complex systems of competing goals which coexist and simultaneously act in the workplace, and their impact on organizational performance depends on their relative and mutual organizational priorities shared by managers and employees [55]. However, risky behaviors under high contextual job demands are likely to increase [56,57], because employees that undertake shortcuts and deviate from safety rules may legitimize these strategies to better meet productivity goals (e.g., [48,58,59]). Therefore, an imbalance between production demands and safety requirements may have serious consequences for employees’ physical health [60]. Moreover, employees tend to share the belief that highly productive workers are more desirable than safety-oriented workers [61], and this may lead employees to share common mental models implicitly accepting the need to systematically violate safety rules to reach productivity goals (e.g., [62]).

In this sense, work pressure reflects a broad concept incorporating a plethora of narrow constructs concerned with employees’ perceptions of different aspects of job demands and their ability to cope with such demands [63,64]. Accordingly, high levels of work pressure can lead to excessive efforts to achieve production goals (e.g., [65]). This set of job demands intrinsically exists in every profit-based organization, since in most cases the ultimate purpose of companies is achieving profit goals.

While sources and effects of production pressure have been studied at the employee level (e.g., [18]), there is theoretical and empirical evidence that such individual perceptions may coalesce at higher levels of analysis. In our current study, we focus on the shared perception among employees within organizations about production pressure and, accordingly, operationalize production pressure climate at the organizational level. In particular, employee perceptions of production pressure may be consistently shaped by the organizational context [66], since individuals are generally exposed within the workplace to common organizational conditions of work pace (e.g., time schedules) and production boundaries (e.g., productivity goals and deadlines). 

It is important to note here that organizational production pressure and safety climates should not be viewed as two sides of the same coin. Indeed, as suggested by Zohar [67], safety climate “should be operationalized in the context of other competing task domains” (p. 1518). In fact, production pressure and safety climate pursue two competing operational goals (i.e., safety versus production) which are not necessarily interdependent. For example, some organizations may prioritize both production and safety aspects (or just one of them), and while employees may espouse a given relative priority (e.g., safety) at the same time they might enact behaviors which are not aligned with it, depending on the kinds of behaviors that maximize the likelihood to be rewarded by the organization [67]. In this vein, Jiang and Probst [20] found evidence for the emergence of a productivity climate, namely the “employees’ shared perceptions of the policies, practices and procedures that are rewarded, supported and expected concerning productivity” (p. 176) at the work-group level. Similarly, we expect individual perceptions of production pressure to be partially shaped by a common organizational core, reflecting the shared organizational tendency to pursue productivity goals at the expense of personal safety (i.e., production pressure climate). Moreover, a higher production pressure climate is predicted to increase the likelihood of circumventing safety rules (e.g., [29,52]) by enacting risky patterns of behavior in order to meet productivity requirements [68]. Overall, these considerations lead us to hypothesize that:

**Hypothesis** **4.** *A higher organizational-level production pressure climate increases the likelihood of employees’ enactment of risky safety behaviors.*

As with safety climate, we not only expect a cross-level direct effect of production pressure climate on individual risky safety behaviors, but also a cross-level moderation effect. Specifically, consistent with the JD-R model, job demands may accumulate and “interact with each other” [1](p. 278). Focusing on our hypothesized model, production pressure climate plays a dual role. On the one hand, it can increase the enactment of individual risky safety behaviors beyond the effect of individual job demands (i.e., workload) and organizational resources (i.e., safety climate). On the other hand, a higher production pressure climate is likely to exacerbate the effect of workload on the enactment of risky safety behavior at the employee level. With regards to the latter, organizations that require employees to work quickly and to systematically achieve stringent deadlines are more prone to normatively promote individual behaviors directed at the pursuit of productivity goals at the cost of safety [69]. This may boost the effect of individually perceived job demands on safety-oriented behaviors by enhancing mechanisms of social comparison and compliance with organizational requirements [70]. Thus, we expect that:

**Hypothesis** **5.** *A higher organizational production pressure climate significantly exacerbates the impact of individually perceived workload on the enactment of risky safety behaviors.*

#### 2.3.2. The Interplay among Organizational Safety and Production Pressure Climates in Relation to Safety Behaviors

Following the typology proposed by Kuenzi and Schminke [11], organizational safety and production pressure climates are focused on core operations, namely “operational goals of the organization” (p. 693). In this respect, each organizational facet-specific climate has its own set of operational goals, which can be conceptualized as their typical facet-specific outcomes. On the one hand, organizational safety climate has been linked to a plethora of safety indicators, such as safety compliance, accidents, injuries and near misses [71]. On the other hand, facet-specific outcomes of organizational production pressure climate may be linked to task performance, such as individual productivity [72].

Although our overarching model explicitly posits a facet-specific outcome of the organizational safety climate (i.e., risky safety behaviors), it is not uncommon that organizations simultaneously prioritize both safety and production policies, practices and rewarded behaviors [73]. From an organizational standpoint, a marked imbalance between safety climate resources and production pressure demands may be interpreted by the employees as a signal of their incompatibility. Indeed, in cases as such these, facet-specific climates “compete for workers’ attention and workers will have to make choices about where to allocate available attention and effort” [48] (p. 301).

In a similar vein, Quinn and Rohrbaugh [74] proposed the competing values framework (CVF), which allows one to distinguish organizational facet-specific climates (i.e., individual or organizational means) and outcomes (i.e., individual or organizational ends) according to their focus and structure (and their possible combinations). With regards to structure, both means and ends can be internal (e.g., focused on the management of employees within the organization) or external (e.g., focused on the dynamics concerned with outer stakeholder, such as customers and clients). With respect to structure, both means and ends can be oriented towards control (e.g., maintenance of stability and internal consistency of organizational practices and processes) or flexibility (e.g., efficiency and productivity of organizational practices and processes).Consistent with the CVF framework, safety climate can be considered an organizational resource (in CVF terms, an organizational means) located within the internal control quadrant, focused on organizational policies, practices and rewarded behaviors aimed at maintaining the homeostasis of employees’ safety which, in turn, can be defined within the CVF framework as an internal control end. Indeed, safety behaviors are enacted by the employees within the organization in compliance with safety rules. Noteworthy, organizational safety climate and individual safety behaviors lie beneath the same CVF quadrant. In contrast, organizational production pressure climate can be assumed as an external flexible means aimed at satisfying efficiency and productivity needs of outer stakeholders and final customers.

Given these premises, since organizational safety and production pressure climates compete for different operational goals [52], one can argue that safety climate may also restrain the impact of other potential threats to individual safety in order to guarantee the internal consistency of the internal control means-ends relationship. Moreover, since safety behaviors are facet-specific outcomes of organizational safety climate [11], we expect this organizational facet-specific climate to moderate the effect of other facet-specific climates focused on alternative competing values (e.g., [20]). In other words, we expect that:

**Hypothesis** **6.** *A higher organizational safety climate significantly attenuates the impact of organizational production pressure climate on the enactment of risky safety behaviors.*

### 2.4. The Present Study

**Figure 1:**
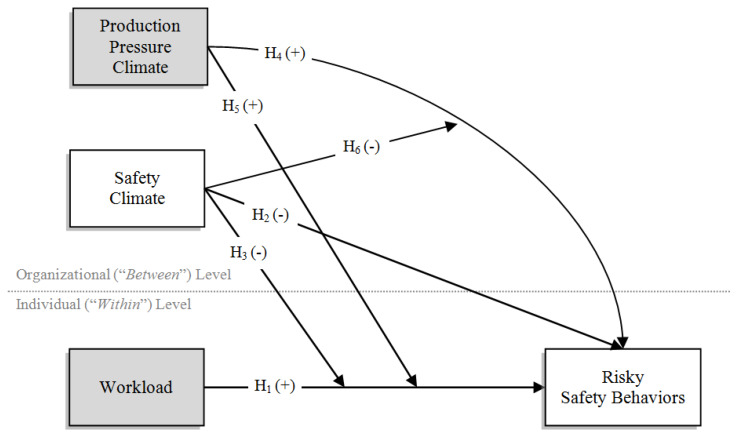
The Overarching Conceptual Model.

Given these premises, the present study aimed at testing the nomological network depicted in Figure 1 within the multilevel framework (exogenous demands are presented as grey boxes, while exogenous resources are presented as white boxes). As illustrated above, we expect that an increase in individual job demands (i.e., workload) may increase the likelihood of enacting unsafe behaviors within the organization (H_1_). Moreover, we expect that such relationship may be mitigated by organizational safety specific climate (H_3_) or exacerbated by organizational production pressure climate (H_5_). Moreover, we also expect that such facet-specific organizational climates may exert a direct influence on risky safety behaviors. Specifically, while organizational safety climate may hinder the enactment of such behaviors (H_2_), we posit that organizational production pressure climate may directly affect them (H_4_). Finally, we expect that organizational safety climate may mitigate the negative effect of organizational production pressure on risky safety behaviors (H_6_).

## 3. Materials and Methods

### 3.1. Participants and Procedure

The present study is rooted within a broader cross-cultural research project investigating the individual and organizational determinants of employee well-being and safety in the United States and Italy. Convenience sampling was applied for both organizations and employees. In order to test our hypotheses, the research team approached administrators and safety representatives of different organizations to probe their interest in the present research project. Once organizations agreed to participate and allow their employees to be recruited, the research team provided information sessions with the employees in order to broadly explain the research topics and to administer questionnaires. Employees signed an informed consent previously approved by the Ethics Committee of the first author’s University Department, which explicitly conveyed the voluntary and anonymous nature of the employees’ participation. Paper-and-pencil questionnaires were distributed to employees and, in most cases, were completed that same day. A small proportion of the sample delivered completed surveys a few days later. The overall response rate at the individual-level was 65–70% of the employees working in the enrolled organizations.

The final sample consisted of N*_i_* =1,375 employees (83.6% male) with a mean age of 41.33 years (SD = 10.34), and with an average tenure in their current job position of 12.13 years (SD = 9.65). The vast majority of the final sample (89.8%) consisted of full-time employees; while the remaining part comprised contingent workers (e.g., temporary, fixed-term, or other non-permanent job contracts). Employees were nested within N*_j_* = 33 small or medium-sized organizations covering a wide array of economic and industrial sectors: distribution/service (n*_j_* = 8), construction (n*_j_*= 7), transportation (n*_j_* = 5), energy production and distribution (n*_j_* = 4), manufacturing (n*_j_* = 4), military, public and private defense (n*_j_* = 3), and health care (n*_j_* = 2). Employees surveyed ranged from 5 to 134 per organization (M = 41.67, SD = 37.65).

### 3.2. Measures

#### 3.2.1. Risky Safety Behaviors

Individual-level risky safety behaviors were measured by a 6-item scale developed by Rundmo [75] intended to capture the frequency of occurrence of risky safety behaviors enacted by employees. Items include typical working activities which can undermine employee physical safety (e.g., “Ignore safety regulations”, “Take risks to complete work tasks”, “Fail to use protective equipment”) and were presented with a five-point Likert format ranging from 1 (“Never or almost never”) to 5 (“Very often or always”).

#### 3.2.2. Workload

Individual workload was assessed by the quantitative workload inventory (QWI) developed by Spector and Jex [26], measuring the amount of work and work pace perceived by the employees. This scale comprises five items (e.g., “How often does your job require you to work very fast?”, “How often does your job require you to work very hard?”) using the same 5-point Likert response scale described above.

#### 3.2.3. Organizational Safety Climate

The 16-item Safety Climate Scale by Neal et al. [9] was used as a measure of organizational safety climate and has been previously validated in the Italian context [76]. These items are grouped into four first-order dimensions, reflecting specific aspects of the overall employees’ perceptions towards the value of safety within the workplace: (1) safety management values (4 items, e.g., “Management considers safety to be important”); (2) safety communication (5 items, e.g., “There is sufficient opportunity to discuss and deal with safety issues in meetings”), (3) safety training (4 items, e.g., “Employees receive comprehensivetraining in workplace health and safety issues”); and, (4) safety systems (3 items, e.g., “Safety procedures and practices are sufficient to prevent incidents occurring”). Items were endorsed on a seven-point Likert-type scale, ranging from 1 (“Strongly disagree”) to 7 (“Strongly agree”).

#### 3.2.4. Organizational Production Pressure Climate

The Organizational Production Pressure Scale [18] was used to assess production pressure climate. This scale is comprised of five items (e.g., “The main focus of this organization is on production—Everything else is secondary”) endorsed with the same 7-point Likert-type format of the safety climate scale. Aggregation of individual safety and production pressure climate scores to the organizational level will be discussed in the following sections.

The full list of the items used for the present study is available as Table S1 of Supplementary Materials.

### 3.3. Analytic Strategy

First, the overall measurement model of the study measures was tested at the individual level. Specifically, we tested a four-factor oblique confirmatory factor model positing four latent dimensions: risky safety behaviors, workload, and safety climate and production pressure (i.e., the constructs implied in the present study). Since individual responses were clustered within organizations, parameters’ standard errors and test statistics were appropriately corrected by taking into account the complex structure of the data [77]. The overall fit of the hypothesized model with the observed data was evaluated with commonly used indices [78] in order to evaluate potential biases due to common method. The hypothesized model was also statistically compared with an alternative confirmatory factor model where an orthogonal common method factor was added to the posited substantive model (i.e., unmeasured common method factor approach [79]).

Next, we evaluated whether it was justifiable to aggregate individual responses regarding safety climate and production pressure to the organizational level using a direct consensus approach [80,81]. Following recommendations by Bliese [82], we first evaluated organizational effects on individual scores with a one-way ANOVA. Second, we calculated two intraclasscorrelation coefficients (ICCs). Specifically, the ICC_(1)_ expresses the proportion of individual scores attributable to such organizational effects [83], while the ICC_(2)_ provides a reliability estimate of organizational-level mean scores. Third, we evaluated the design effect (*deff*, [84]) to evaluate the presence of clustering effects in the sampling structure of the data. Finally, we also computed the rwg_(*j*)_ [85] assuming *a priori* a rectangular distribution of the agreement [86] as a measure of within-organization agreement of individual-level scores. Based on established recommendations, we used the following cut-off values to determine the appropriateness of the aggregation procedure: (a) an ICC_(1)_ ≥ 0.10 [87]; (2) an ICC_(2)_ ≥0.60 [88,89]; (c) a *deff* ≥ 2 [84]; (4) an rwg_(*j*)_ reflecting at least moderate within-organization agreement (>0.50, [90]).

Reliability estimates were calculated differently for individual-level scores (i.e., workload) and for the other aggregated measures (i.e., organizational safety and production pressure climates). Since risky safety behaviors are supposed to vary both across individuals and organizations, both types of estimates were calculated and examined. Following recommendations [91], three indices were taken into account: (1) Cronbach’s α, providing information about internal consistency among the items of a given scale; (2) composite reliability (*ω*), which can be interpreted as the degree of internal consistency when the tau-equivalence assumption among the items is released, and the (3) maximal reliability (*H*), which can be interpreted as the reliability about the scale’s optimally weighted composite. Given the relatively small number of organizations considered in the present study, parameter estimates for multilevel reliability coefficients were derived by a series (one per measure) of multilevel confirmatory single-factor models analyzed through Bayesian estimation with uninformative priors [92,93].

We next tested our hypothesized multilevel model presented in Figure 1 using the steps recommended by Heck and Thomas [84] using maximum likelihood estimators with robust standard errors (MLR, [94]) with the full information maximum likelihood (FIML) to handle missing data. It is important to note that, although multilevel regression coefficients are generally unbiased even under conditions of small level-2 (i.e., organizational) sample sizes, standard errors of variance components may display relevant downward biases [95]. Accordingly, given our limited organizational-level sample size (i.e., N*_j_* = 33), the use of MLR estimators is warranted, since it provides more accurate estimates of variance parameters than other procedures (e.g., the use of maximum likelihood with asymptotic standard errors [96]).

We tested our hypotheses using a step-by-step approach [83,84]. Although given the limited number of organization involved in the study these models were tested using observed composites of the administered scales rather than latent variables, such piecewise approach takes important advantages over other analytical techniques (e.g., pooled regression approach). Specifically and conceptually similar to classical hierarchical regression models, it allows for a step-by-step evaluation of the statistical significance of the outcomes’ variance components at both levels after the introduction of new predictors. Moreover, such approach allows to determine progressively the added contribution of organizational-level variable (and their interaction) in explaining both the outcome variable and the organizational random component of the individual-level direct effect.

First, the “null model” (Model 1) was tested. This model posits no predictors of the outcome (i.e., risky safety behaviors) and allows for the decomposition of the outcome variance into its components attributable, respectively, to individual and organizational levels. With regards to the latter, this should be significantly different from zero in order to meaningfully conduct the subsequent multilevel analysis [97]. After establishing that a statistically significant part of risky safety behaviors variability is attributable to differences between organizations, the individual-level predictor (i.e., workload) can be included (Model 2, also known as “random-intercept model”). In this model, it is important to note that the effect of workload on risky safety behaviors is specified as fixed and does not vary across organizations. In Model 3 (i.e., the “intercepts-as-outcome” model) organizational safety and production pressure climates are added to the former model as organizational-level predictors of risky safety behaviors. In contrast to the effect of workload on the individual-level part of risky safety behaviors’ variability, organizational-level independent variables exert their direct effect on the organizational-level counterpart of risky safety behaviors. In Model 4, generally known as “intercepts-as-outcome with random slopes” model, the individual-level effect of workload on risky safety behaviors is free to vary across different organizations (i.e., individual-level slopes are random across organizations). This allows for the evaluation of the statistical significance of the variability around the random slopes term between organizations. After this check, Model 5 (the so-called “intercepts- and slopes-as-outcomes model”) was carried out. This model tests simultaneously all the hypotheses (excepting H_6_) depicted in Figure 1, where the organizational-level variables are expected to explain the variability of the random slopes (i.e., cross-level interaction terms). Finally, Model 6 adds to the previous model the interactive effect among organizational safety and production pressure climate for the explanation of risky safety behaviors.

Adjacent nested models (e.g., Model 2 vs. Model 1) were compared using the differences between their −2log-likelihood (−2LL), which is distributed as a chi-square [83]. Finally, in order to avoid interpretation biases, the individual-level predictor was group-mean centered, while organizational-level independent variables were grand-mean centered [98].

## 4. Results

### 4.1. Overall Measurement Model

The overall measurement model was estimated at the individual level using robust maximum likelihood estimators and correcting parameters’ standard errors for the non-independence of the observations by using the “TYPE = COMPLEX” command in Mplus 8.4 [94], which provides a corrected chi-square which is asymptotically equivalent to the Yuan–Bentler T2* test statistic [99]. The hypothesized four-factor model fitted satisfactorily the observed data: YBχ^2^_(1,375; *df*=454)_ = 1580.15, < 0.001; root mean squared error of approximation (RMSEA) = 0.042 (90% confidence interval 0.040−0.045); comparative fit index (CFI) = 0.959; Tuker–Lewis or Non-Normed Fit Index (TLI or NNFI) = 0.955; SRMR = 0.056. When adding the orthogonal common method factor, the overall model fit was: YBχ^2^_(1375; *df*=422)_ = 1322.16, *p* < 0.001; RMSEA = 0.039 (90% confidence interval 0.037−0.042); CFI = 0.967; TLI = 0.961; SRMR = 0.028. Since the ∆CFI [100] between our substantive (more parsimonious) measurement model and the model adding the common factor was 0.008 (i.e., <0.01), we can conclude that the originally hypothesized 4-factor model does not fit worse than the confirmatory factor model positing the additional common method factor. This finding supports that responses to the items vary as a function of their referent substantive latent constructs rather than common method artifacts.

### 4.2. Aggregation Procedures

Safety climate scores varied significantly across the sampled organizations: *F*(32,1341) = 18.89, *p* < 0.001. The ICC_(1)_ was 0.314, while the ICC_(2)_ was 0.951, while the design effect (*deff*) was 13.769. The rwg_(16)_ assuming a rectangular distribution of the agreement was 0.93 (SD = 0.06). With regards to production pressure, individual scores also significantly varied across organizations: *F*(32,1336) = 18.89, *p* < 0.001. The ICC_(1)_ was 0.152, while the ICC_(2)_ was 0.883, while the design effect (*deff*) was 7.364. The rwg_(5)_ assuming a rectangular distribution of the agreement 0.57 (SD = 0.27). Overall, these findings highly support the aggregation of individual safety climate and production pressure scores at the level of the organizations, and such aggregates may be legitimately interpreted as organizational safety and production pressure climates, computed as within-organization mean scores.

### 4.3. Descriptive Statistics of the Study Variables

Table 1 presents descriptive statistics of the study variables. As can be noted, the distribution of all variables conforms to univariate normality, although risky safety behaviors showed a moderate negative skewness at both levels of analysis. Reliability level-specific coefficients were high in all cases, suggesting that observed scores are only weakly biased by random measurement error. At the individual level, risky safety behaviors were positively correlated with workload (*r* = 0.32, *p* < 0.01) and production pressure (*r* = 0.37, *p* < 0.01), while a negative correlation was observed with safety climate (*r* = −0.41, *p* < 0.01). A weak negative correlation was also observed among workload and safety climate (*r* = −0.19, *p* < 0.01), which in turn was negatively correlated with production pressure (*r* = −0.17, *p* < 0.01). Finally, a weak positive correlation was observed among workload and production pressure scores (*r* = 0.16, *p* < 0.01). At the organizational level, the pattern of correlations was similar, with most of coefficients showing a higher magnitude than those observed at the individual level. However, the correlation between workload and organizational safety climate was not significant. Moreover, the correlation between safety climate and production pressure climate was also non-significant, further bolstering the contention that these are separate and unique facets of an organization’s climate.

### 4.4. Multilevel Modeling

Results from the tested multilevel models are shown in Table 2. In Model 1, it can be noted that risky safety behavior scores significantly varied across organizations (u_0_ = 0.160, *p* < 0.001). Specifically, 19.4% of the variability around risky safety behaviors depended on organizational level differences. The random-intercept model (Model 2) showed a positive regression coefficient of workload on risky safety behaviors at the individual level (B_1_ = 0.310, *p* < 0.001), explaining 9.5% of the outcome variability located at the individual level. This finding is consistent with the hypothesized individual-level direct effect (H_1_). The intercepts-as-outcome model (Model 3) showed a negative effect of organizational safety climate (γ_01_ = −0.197, *p* < 0.001) and a positive effect of organizational production pressure climate (γ_02_ = 0.439, *p* < 0.001) on the organizational level part of risky safety behaviors. Such effects, which were consistent with hypotheses H_2_ and H_4_, explained 67.5% of the random intercepts.

In Model 4, the random slope coefficient was found to statistically differ from zero (u_1_ = 0.041, *p* < 0.001), suggesting that the effect of workload on risky safety behaviors significantly varies as a function of organizational differences, which were significantly explained by organizational climates’ cross-level interactive effects (as shown in Model 5). Specifically, organizational safety climate showed a negative (buffering) effect on the random slopes (γ_11_ = −0.097, *p* < 0.01), while a positive (exacerbating) effect was found for organizational production pressure climate (γ_12_ = 0.211, *p* < 0.001). Thus, consistent with H_3_ and H_5_ expectations, both organizational safety and production pressure climates significantly explain organizational differences related to the effect of workload on risky safety behaviors in the expected direction. Overall, these cross-level effects explained 63.4% of the overall variability of the random slopes. Interestingly, after including the organizational level moderators, the variability around the random slopes was no longer significant (u_1_ = 0.015, *p* > 0.05).

**Figure 2:**
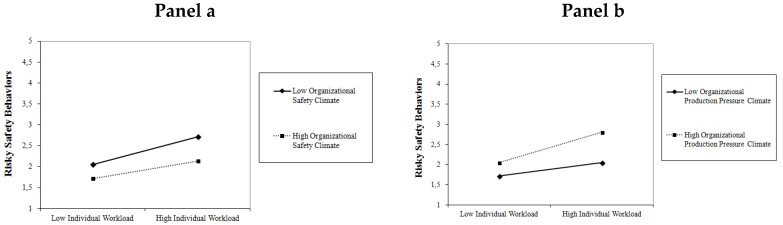
Plots of Cross-Level Interactions from the Final Multilevel Model.

Plots of the cross-level interactions are presented in Figure 2. As can be seen, on the one hand, a positive organizational safety climate mitigates the impact of workload on risky safety behaviors, especially under conditions of high workload (see Panel a). On the other hand, a high organizational production pressure climate exacerbates the individual level effect, especially under conditions of high workload (see Panel b).

**Figure 3:**
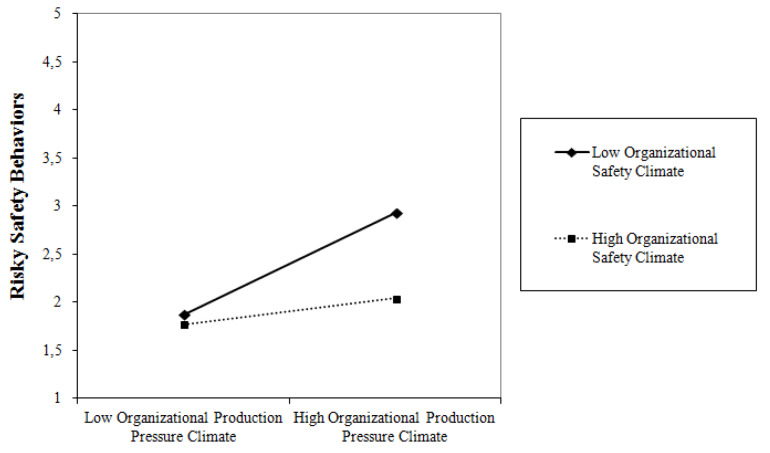
Plots of the Interaction Among Safety and Production Pressure Climates from the Final Multilevel Model.

Finally, Model 6 highlighted a significant cross-level effect of the interaction among organizational climates over risky safety behaviors (γ_21_ = −0.324, *p* < 0.01), which is in line with H_6_ expectations. In this final model, organizational safety and production pressure climates and their interaction explained the 75.6% of the random intercepts of risky safety behaviors scores, with the interactive effects adding a unique contribution of 8.1% of explained variance above and beyond main cross-level effects. As showed in Figure 3, a high safety climate hinders the effect of production pressure climate in enhancing the enactment of risky safety behaviors, especially when production pressure climate is high.

## 5. Discussion

The present study responds to recent calls aimed at investigating the impact of job demands and resources on relevant job-related outcomes within a multilevel framework [1]. Specifically, the purpose of the present study was to explain employees’ decision to enact risky safety behaviors by testing a multilevel model positing: (1) the impact of individual-level demands (i.e., workload); (2) the cross-level effects of multiple competing organizational climates (i.e., safety and production pressure climates); (3) the cross-level interactive effects of such facet-specific climates on the individual job demands-safety link; and, (4) the impact of the interaction among these competing climates in explaining our safety outcome. To test these propositions, we collected data from employees nested within different organizations distributed across several economic sectors.

At the individual level, the impact of workload on risky safety behaviors was significant and in the expected direction (H_1_). This finding suggests that employees experiencing a higher workload (e.g., an excessive volume of work) are more prone to enact behaviors which are not in line with safety requirements. This effect may be interpreted as the signal of selective and compensatory processes leading employees to protect their expected performance level by reallocating their personal resources to the detriment of workplace safety [38,101]. This is also consistent with the safety-production compatibility principle, namely “the degree to which safe work is complementary to accomplishing a production task” [48](p. 301). Therefore, it may be argued that a high workload may undermine the safety-production balance by breaking this principle, leading employees to undertake risky safety behaviors in order to instrumentally allocate their finite personal resources towards tasks which respond to organizational productivity expectations [52].

In accordance with the view that multiple climates coexist in organizational settings [11] and that both safety- and production-oriented priorities influence organizational [56], we examined the dual impact of organizational safety and production pressure climates and their cross-level interactive effects with workload in explaining risky safety behaviors. With regards to organizational safety climate, the hypothesized direct (H_2_) and interactive (H_3_) effects on the workload-risky safety behaviors relationship were supported by the study findings. These findings are consistent with a large body of prior knowledge supporting that organizational safety climate may promote safety compliance (e.g., [6]) and may mitigate the strength of the impact of excessive job demands on safety behaviors (e.g., [44]). With respect to organizational production pressure climate, the expected direct (H_4_) and interactive (H_5_) effects were also supported by the study findings. Specifically, this organizational variable was directly and positively associated to risky safety behaviors. This finding is consistent with research suggesting that an organizational climate focused on productivity may increase the likelihood that employees experience safety and productivity as distinct operational goals competing for the same individual resources [20,52]. Moreover, our results suggest that a high organizational production pressure climate may exacerbate the impact of excessive job demands on undesirable safety outcomes. In other words, under conditions of high workload experienced by the employees, organizational production pressure climate may increase the salience of several productivity aspects (e.g., the pace of work and work deadlines) which, in turn, may promote the enactment of risky safety behaviors. This finding is also consistent with the fact that, under excessive job demands, employees are more prone to emphasize productivity priorities as a signal allowing unsafe shortcuts in order to maintain a homeostatic level of job performance [37].

Moreover, organizational safety climate mitigates the cross-level impact of organizational production pressure climate on the enactment of risky safety behaviors (H_6_). This finding is consistent with the arguments provided by the competing climate hypothesis [23] and the competing values framework [74]. With this regard, it may be argued that organizational safety climate is likely to protect its facet-specific outcome (i.e., individual safety) from potential organizational threats (i.e., production pressure climate) in order to guarantee a relative balance between competing means (i.e., competing face-specific climates) pointing to different operational goal systems (i.e., safety versus production, [52]), which however draw on common finite resources [101].

### 5.1. Theoretical Implications

The present study responded to recent calls to extend the JD-R framework to a multilevel context [1] by explicitly acknowledging that demands and resources can accrue at multiple levels and influence the enactment of employee safety behaviors. From a theoretical standpoint, the present findings may have relevant implications.First, the JD-R model may be used as a promising theoretical framework to understand safety-related individual behaviors through a multilevel organizational lens. In this sense, our results suggest that organizational demands and resources may play a dual role: on the one hand, they can contribute in uniquely explaining individual safety outcomes while, on the other hand, they may exacerbate (or buffer) the effects of individual demands on safety performance. Moreover, our results also highlighted the potential importance of considering the interplay among organizational demands and resources in explaining individual safety-related behaviors.

Second, we supported both theoretically and empirically that production pressure perceptions may not be attributable exclusively to individual differences, but a relevant part of such perceptions may be shared within the organization. In line with prior research in the field [20], the present study stressed that production pressure can be characterized as an important organizational demand and meaningfully operationalized as a climate variable.

Third, the present study supported the view that multiple climates may coexist within the organization [11]. With this regard, our findings suggest that organizational safety and production pressure climates may be conceived as relatively independent constructs, which coexist within the organization by competing for different operational goals [52] but drawing on common personal resources (e.g., effort and attention [48]).

### 5.2. Practical Implications, Limitations and Future Directions

Our study findings also suggest some relevant implications for practice. At the level of the employees, experiencing a high workload may lead to serious consequences for their personal safety by engaging in risky behaviors and circumventing safety rules to maintain the requested level of performance standards. Organizational policies and job design practices should take this into account by planning achievable deadlines and accurately distributing the amount of work among employees joining common production goals. At the organizational level, managers may acknowledge that multiple competing climates can coexist simultaneously, and they should not be considered as mutually exclusive when planning interventions to promote either safety or productivity. Indeed, although safety and production pressure climates only demonstrated a weak association, our results clearly showed that both may act simultaneously as: (1) determinants of individual risky safety behaviors; (2) moderators of the effects of individual-level job demands on such behaviors; and, (3) interacting at the organizational level in shaping individual safety behaviors. Our results suggest that organizational-level interventions aimed at promoting individual safety behaviors within the workplace may enhance the safety-production compatibility principle [48] by emanating unequivocal signals that safety compliance should not be compromised in favor of productivity. As such, explicit verbal communication based on specific coaching programs for organizational managers and leaders can be used as a leverage to modify shared employee climate perceptions [102] and to reduce potential imbalance between safety and production pressure climates.

Despite these implications, some limitations of the present study should be acknowledged. First, our results are based on self-reported data. Further research should collect data from multiple sources and informants (e.g., number of known enacted risky behaviors and their associated disciplinary measures may be derived from human resources archival data). Second, organizations and employees were enrolled with a snowball sampling strategy within a single national context; as such, the generalizability of the study findings should be strengthened with additional replication studies within a cross-cultural perspective. Moreover, the study sample comprised a very large percentage of males (83.6% of the total). Finally, the sampled organizations were rather heterogeneous and in a limited number. Further research efforts should point to replicate our study findings by considering a larger number of organizations nested within specific economic and industrial sectors (e.g., high-risk and high-performance industries). Moreover, the present findings should be replicated by considering more heterogeneous samples in terms of gender. Further studies may also consider the inclusion of an additional meso-level of analysis (i.e., group-level) in order to better understand the relationship among job demands and resources within the multilevel framework, including the influence of workgroup-level variables such as supervisory safety leadership.

## 6. Conclusions

The present study adds unique knowledge about the explanation of safety-oriented behaviors by highlighting the importance of considering multiple organizational climates as relevant determinants. Moreover, such organizational features may play important yet distinct roles in moderating the effect of individual job demands on safety outcomes. In the current study, whereas organizational safety climate attenuated the positive relationship between workload and risky safety behaviors, organizational-level production pressure climate exacerbated this relationship. Finally, organizational safety climate mitigated the cross-level effect of organizational production pressure climate on risky safety behaviors. Future studies are encouraged to replicate the present findings across multiple cultural contexts and by considering data from multiple sources including multiple levels of analysis (e.g., work groups and/or or department levels).

## Figures and Tables

**Table 1:** Descriptive Statistics, Reliability Coefficients and Zero-Order Correlations among the Study Variables.

	Descriptive Statistics								Reliability									
	IndividualLevel				OrganizationalLevel				IndividualLevel			OrganizationalLevel			Correlations			
	M	SD	Sk	Kurt	M	SD	Sk	Kurt	α	ω	H	α	ω	H	1.	2.	3.	4.
1. Risky Safety Behaviors	2.10	0.90	0.76	0.00	2.16	0.46	0.87	0.10	0.89	0.89	0.90	0.97	0.98	0.99	−	0.50 **	−0.41 *	0.60 **
2. Workload	3.15	0.86	−0.11	−0.18	3.14	0.41	−0.39	−0.039	0.84	0.84	0.84	−	−	−	0.32 **	−	−0.12	0.38*
3. Safety Climate	4.67	1.26	−0.44	−0.29	4.44	0.77	−0.06	−0.50	−	−	−	0.98	0.96	0.97	−0.41 **	−0.19 **	−	−0.13
4. Production Pressure	3.32	1.34	0.20	−0.61	3.31	0.63	0.30	−0.94	−	−	−	0.91	0.94	0.98	0.37 **	0.16 **	−0.17 **	−

*Note.* M = mean; SD = standard deviation; Sk = skewness; Kurt = kurtosis; α = level-specific Cronbach’s alpha; ω = level-specific composite reliability; H = level-specific maximal reliability. ** *p* < 0.01, * *p* < 0.05. Correlations below the diagonal refers to individual level, those above the diagonal pertain to organizational level.

**Table 2:** Estimated Coefficients from the Tested Multilevel Models.

	Model 1	Model 2	Model 3	Model 4	Model 5	Model 6
**Fixed Part**	**Coeff. (s.e.)**	**Coeff. (s.e.)**	**Coeff. (s.e.)**	**Coeff. (s.e.)**	**Coeff. (s.e.)**	**Coeff. (s.e.)**
γ_00_	2.147 (0.057) ***	2.149 0(.078) ***	2.154 (0.048) ***	2.154 (0.048) ***	2.154 (0.048) ***	2.140 (0.046) ***
B_1_ Workload		0.310 (0.053) ***	0.310 (0.053) ***	0.318 (0.049) ***	0.334 (0.036) ***	0.334 (0.036) ***
γ_01_ Safety Climate			−0.197 (0.060) ***	−0.197 (0.061) ***	−0.197 (0.061) ***	−0.306 (0.074) ***
γ_02_ Production Pressure Climate			0.440 (0.096) ***	0.439 (0.097) ***	0.439 (0.097) ***	0.439 (0.082) ***
γ_11_ Workload * Safety Climate					−0.097 (0.039) **	−0.097 (0.039) **
γ_12_ Workload * Production Pressure Climate					0.211 (0.056) ***	0.211 (0.056) ***
γ_21_ Safety Climate * Production Pressure Climate						−0.324 (0.036) **
**Random Part**						
u_0_	0.160 (0.043) ***	0.163 (0.043) ***	0.051 (0.021) *	0.052 (0.022) *	0.052 (0.022) *	0.039 (0.011) *
u_1_				0.041 (0.013) ***	0.015 (0.013)ns	0.015 (0.013)ns
r	0.664 (0.057) ***	0.600 (0.040) ***	0.601 (0.040) ***	0.574 (0.037) ***	0.576 (0.037) ***	0.575 (0.037) ***
−2*LogLikelihood (*NeP*)	3390.18 (3)	3254.35 (4)	3228.58 (6)	3197.04 (7)	3186.10 (9)	3179.48 (10)
∆−2*LogLikelihood (*df*)		135.83 (1) ***	25.77 (2) ***	31.54 (1) ***	10.94 (2) **	6.63 (1) *

*Note*. Model 1 = null model; Model 2 = random intercept model; Model 3 = intercepts-a-outcome model; Model 4 = intercepts-as-outcome model with random slopes; Model 5 = Intercepts and slopes-as-outcomes model; Model 6 = Intercepts and slopes-as-outcomes model plus cross-level effect of the interaction among safety and production pressure climates on risky safety behaviors. Coefficients (Coeff.) and standard errors (s.e.) refers to the unstandardized solution. NeP = number of estimated parameters. *** *p* < 0.001, ** *p* < 0.01, * *p* < 0.05, *ns* = non-significant.

## Supplementary Materials

The following are available online at https://www.mdpi.com/1660-4601/17/10/3496/s1, Figure S1: Flow Chart of the Study Process, Table S1: List of Items Used for The Study Measures.
