# Supplementary material for: Multilevel Job Demands and Resources: Cross-Level Effects of Competing Organizational Facet-Specific Climates on Risky Safety Behaviors
Source: Int J Environ Res Public Health. 2020 May 17;17(10):3496. doi: 10.3390/ijerph17103496 (PMC7277467; doi:10.3390/ijerph17103496)
Supplement: Supplementary file 1 [file ijerph-17-03496-s001.pdf]

Supplementary Materials

Figure S1. Flow Chart of the Study Process.

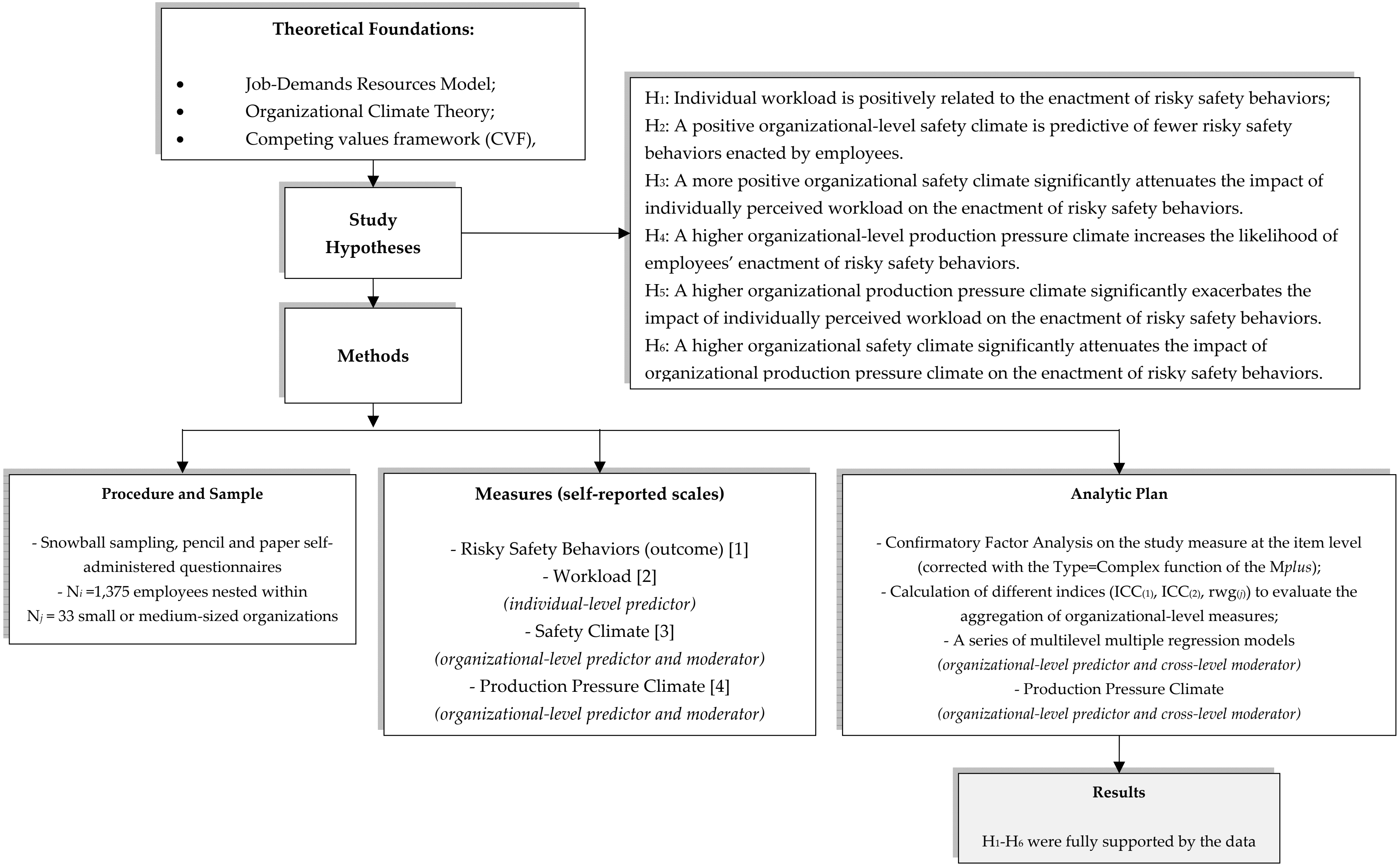

Table S1. List of Items Used for The Study Measures.

**Risky Safety Behaviors** [1]
1. Ignore safety regulations
2. Carry out forbidden activities
3. Cannot always perform work correctly
4. Take chances to get job done
5. Do not use protective equipment
6. Break procedures
**Workload** [2]
1. How often does your job require you to work very fast?
2. How often does your job require you to work very hard?
3. How often does your job leave you with little time to get things done?
4. How often is there a great deal to be done?
5. How often do you have to do more work than you can do well?
**Safety Climate** [3]
1. Management is concerned for the safety of its employees
2. Management places a strong emphasis on workplace health and safety
3. Safety is given a high priority by management
4. Management considers safety to be important
5. There is frequent communication about safety issues in your organization
6. Employees are able to discuss their concerns about safety issues with management
7. There is sufficient opportunity to discuss and deal with safety issues in meetings
8. There is open communication about safety issues within your organization
9. Employees are regularly consulted about workplace health and safety issues
10. Safety issues are given a high priority in training programs
11. Workplace health and safety training cover the types of situations that employees encounter in their job
12. Employees receive comprehensive training in workplace health and safety issues
13. Employees have sufficient access to workplace health and safety training programs
14. Safety procedures and practices are sufficient to prevent incidents occurring
15. There are systematic procedures in place for preventing breakdowns in workplace safety
16. The safety procedures and practices in your company are useful and effective
**Production Pressure Climate** [4]
1. I sometimes need to compromise safety in order to meet production demands
2. My job security is at risk if production demands are not met
3. I may be demoted or experience other negative job-related consequences if production demands are not met
4. I experience workplace injuries due to production demands
5. The main focus of this organization is on production. Everything else is secondary

References

1. Rundmo, T. Associations between risk perception and safety. *Saf. Sci.* **1996**, *24*, 197–209, doi:10.1016/S0925-7535(97)00038-6.
2. Spector, P.E.; Jex, S.M. Development of four self-report measures of job stressors and strain: Interpersonal Conflict at Work Scale, Organizational Constraints Scale, Quantitative Workload Inventory, and Physical Symptoms Inventory. *J. Occup. Health Psychol.* **1998**, *3*, 356–367, doi:10.1037/1076-8998.3.4.356.
3. Neal, A.; Griffin, M.A.; Hart, P.M. The impact of organizational climate on safety climate and individual behavior. *Saf. Sci.* **2000**, *34*, 99–109, doi:10.1016/S0925-7535(00)00008-4.
4. Probst, T.M.; Graso, M. Pressure to produce=pressure to reduce accident reporting? *Accid. Anal. Prev.* **2013**, *59*, 580–587, doi:10.1016/j.aap.2013.07.020.
